# Glycosylation of Erythrocyte Spectrin and Its Modification in Visceral Leishmaniasis

**DOI:** 10.1371/journal.pone.0028169

**Published:** 2011-12-02

**Authors:** Sajal Samanta, Devawati Dutta, Angana Ghoshal, Sumi Mukhopadhyay, Bibhuti Saha, Shyam Sundar, Saulius Jarmalavicius, Michael Forgber, Chhabinath Mandal, Peter Walden, Chitra Mandal

**Affiliations:** 1 Cancer and Cell Biology Division, Council of Scientific and Industrial Research-Indian Institute of Chemical Biology, Kolkata, India; 2 Department of Tropical Medicine, School of Tropical Medicine, Kolkata, India; 3 Department of Medicine, Institute of Medical Sciences, Banaras Hindu University, Varanasi, India; 4 Department of Dermatology, Charité-Universitätsmedizin Berlin, Humboldt University, Berlin, Germany; 5 National Institute of Pharmaceutical Education and Research, Council of Scientific and Industrial Research-Indian Institute of Chemical Biology, Kolkata, India; University of South Alabama, United States of America

## Abstract

Using a lectin, Achatinin-H, having preferential specificity for glycoproteins with terminal 9-*O*-acetyl sialic acid derivatives linked in α2-6 linkages to subterminal *N*-acetylgalactosamine, eight distinct disease-associated 9-*O*-acetylated sialoglycoproteins was purified from erythrocytes of visceral leishmaniaisis (VL) patients (RBC_VL_). Analyses of tryptic fragments by mass spectrometry led to the identification of two high-molecular weight 9-*O*-acetylated sialoglycoproteins as human erythrocytic α- and β-spectrin. Total spectrin purified from erythrocytes of VL patients (spectrin_VL_) was reactive with Achatinin-H. Interestingly, along with two high molecular weight bands corresponding to α- and β-spectrin another low molecular weight 60 kDa band was observed. Total spectrin was also purified from normal human erythrocytes (spectrin_N_) and insignificant binding with Achatinin-H was demonstrated. Additionally, this 60 kDa fragment was totally absent in spectrin_N_. Although the presence of both *N*- and *O*-glycosylations was found both in spectrin_N_ and spectrin_VL_, enhanced sialylation was predominantly induced in spectrin_VL_. Sialic acids accounted for approximately 1.25 kDa mass of the 60 kDa polypeptide. The demonstration of a few identified sialylated tryptic fragments of α- and β-spectrin_VL_ confirmed the presence of terminal sialic acids. Molecular modelling studies of spectrin suggest that a sugar moiety can fit into the potential glycosylation sites. Interestingly, highly sialylated spectrin_VL_ showed decreased binding with spectrin-depleted inside-out membrane vesicles of normal erythrocytes compared to spectrin_N_ suggesting functional abnormality. Taken together this is the first report of glycosylated eythrocytic spectrin in normal erythrocytes and its enhanced sialylation in RBC_VL_. The enhanced sialylation of this cytoskeleton protein is possibly related to the fragmentation of spectrin_VL_ as evidenced by the presence of an additional 60 kDa fragment, absent in spectrin_N_ which possibly affects the biology of RBC_VL_ linked to both severe distortion of erythrocyte development and impairment of erythrocyte membrane integrity and may provide an explanation for their sensitivity to hemolysis and anemia in VL patients.

## Introduction

The erythrocyte membrane is supported by a well-structured cytoskeleton. This cytoskeleton comprises of a network of different proteins maintaining the structural integrity and rigidity of the red blood cell (RBC) and of the RBC membrane [1]. Spectrin is a major cytoskeletal protein present as tetramers of α- and β-subunits associated with other cytoskeletal proteins forming a lattice that governs erythrocyte membrane properties. Alterations of spectrin have been associated with several congenital anomalies like hereditary hemolytic anemia and hereditary elliptocytosis leading to cellular distortion [2]. Biochemical modifications of spectrin, mainly glycation and oxidation, have been observed in diabetes mellitus indicating erythrocyte membrane changes [3]–[4]. Therefore, the status of the cytoskeletal proteins in disease may be affected by genetic abnormalities or metabolic or other stress inducing changes in cytoskeletal protein structure.

Different levels and/or pattern of terminal sialic acid (SA) and its *O*-acetylation of cell surface expressed sialoglycoconjugates have occupied a pivotal position in inducing changes in different diseases [5]–[8]. The presence of different derivatives of SA on *Leishmania donovani* [9]–[13], different immune cells [14], [15] and RBC [16]–[18] of patients with visceral leishmaniasis (VL) (RBC_VL_) and their role [19]–[26] have been demonstrated.

VL caused by the intracellular kinetoplastid protozoa *L. donovani* accounts for an estimated 12 million infected humans with an incidence of 0.5 million cases per year [27]–[28]. Approximately 50% of the world's VL cases occur in the Indian subcontinent. Along with other signature manifestations, VL is almost always associated with anemia [17]–[18]. However alteration of the RBC membrane architecture as one of the causes leading to anemia remains poorly understood.

We have detected the exclusive presence of eight distinct disease-associated 9-*O*-acetylated sialoglycoproteins (9-*O*-AcSGPs) on RBC_VL_ [17], using the preferential specificity of a snail lectin, Achatinin-H for glycoproteins with terminal 9-*O*-acetyl sialic acid (9-*O*-AcSA) derivatives linked in α2-6 linkages to subterminal *N*-acetylgalactosamine (GalNAc) [29]. Interestingly, normal erythrocytes (RBC_N_) are devoid of such 9-*O*-AcSGPs. Antibodies directed against *O*-acetylated sialic acids have also been demonstrated in VL [30], [15]. Moreover enhanced pattern of altered sialylation demonstrated a direct correlation with the degree of complement-mediated hemolysis of RBC_VL_ providing a plausible basis for anemia associated with VL [18]. Taking into consideration the involvement of 9-*O*-AcSGPs in VL erythrocyte pathology, we report the presence, purification and identification of sialylation, *N*- and *O*-glycosylation of two high molecular weight *O*-acetylated sialoglycoproteins as human eythrocytic α and β-spectrin by analysis of the tryptic fragments using matrix-assisted laser desorption/ionization time-of-flight (MALDI-TOF)/post source decay (PSD) mass spectrometry (MS). Purified of spectrin_VL_ showed the presence of an additional 60 kDa band which is completely absent in spectrin_N_ purified by the same procedure. Additionally we have demonstrated glycosylation of spectrin_N_ purified from normal erythrocytes (RBC_N_). Although the presence of *N*- and *O*-glycosylations was found both in spectrin_N_ and spectrin_VL_, enhanced sialylation was largely induced only in spectrin_VL_. Controlled tryptic fragments of α- and β-spectrin_VL_ exhibited the presence of terminal linkage specific sialic acids. This enhanced sialylation is possibly related to the fragmentation of spectrin in VL as evidenced by an additional 60 kDa fragment in spectrin_VL_ and totally absent in spectrin_N_. In summary, this is the first report of glycosylation in eythrocytic spectrin_N_ and its modifications in diseased condition which possibly affects the biology of RBC_VL_ and may provide an explanation for their sensitivity to hemolysis and anemia in VL patients.

## Results

### Eight distinct disease-associated 9-*O*-AcSGPs exclusively induced on RBC_VL_

**Figure 1:**
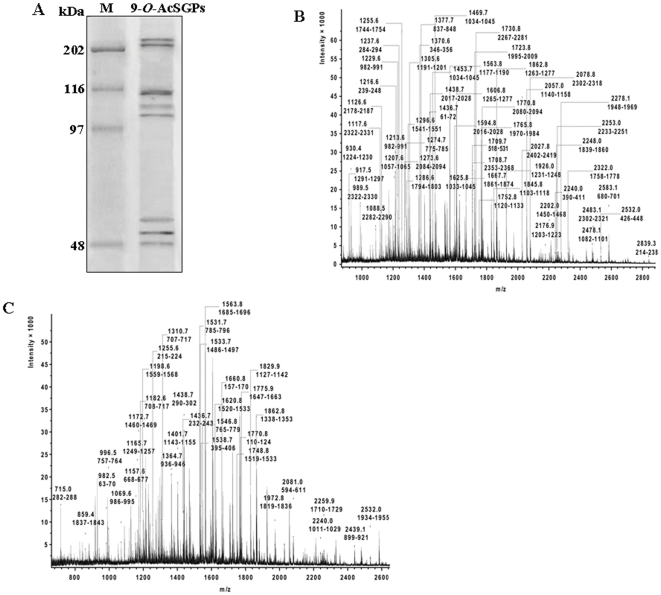
Purification of 9-*O*-AcSGPs and identification of spectrin. ***A.*** A representative SDS-PAGE (7.5%) profile of purified 9-*O*-AcSGPs from RBC_VL_. Lane M shows molecular weight standards. ***B–C.*** PMF spectra of tryptic fragments of two high molecular weight 9-*O*-AcSGPs were identified as α- (B) and β-spectrin (C) by MALDI-TOF MS. Each fragment is denoted by their m/z values and sequence range in human α- and β-spectrin.

Disease-associated 9-*O*-AcSGPs were purified from RBC_VL_ of clinically confirmed untreated VL patients (n = 30) using Achatinin-H-Sepharose 4B affinity matrix (Fig. 1A). Total membrane protein (1.20±0.187 mg) obtained from 2×10^10^ RBC_VL_ yielded 0.432±0.025 mg of purified 9-*O*-AcSGPs separated as eight distinct bands on SDS-PAGE. Purification of the same from RBC_N_ (2×10^10^) of normal healthy individuals (n = 30) by the same procedure yielded undetectable amount of protein.

### Identification of spectrin by mass spectrometry

Molecular identification of VL-associated proteins is a key to their significance in the disease pathology. With this aim, peptide mass fingerprint (PMF) analysis and sequencing of tryptic fragments of the two high molecular weight bands of 9-*O*-AcSGPs by MALDI-TOF/PSD-MS led to the identification of erythrocytic α- and β-spectrin with sequences coverage of 33.6% and 22.7% respectively (Fig. 1B–C). These tryptic fragments were mapped on to the NCBI database sequences of human erythrocytic α- and β-spectrin sequences gi: 119573202 and gi: 67782321 respectively (shown in red color in Fig. S1).

### Spectrin_VL_ is specifically a 9-*O*-AcSGP present only in RBC_VL_

**Figure 2:**
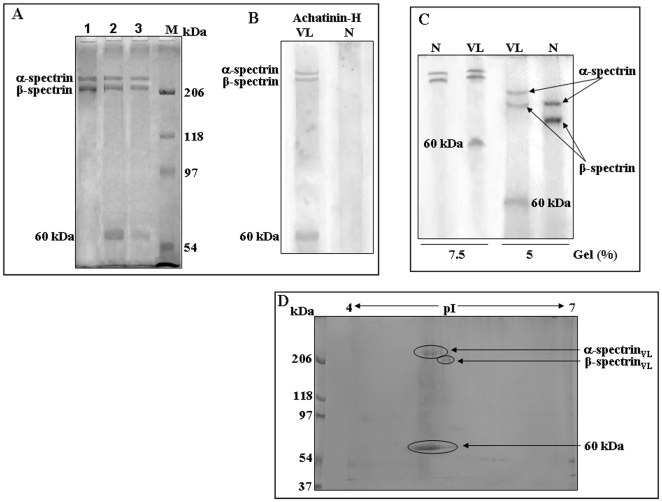
Purification and characterization of spectrin. ***A.*** *Purification of spectrin_VL_ and spectrin_N_.* A representative SDS-PAGE (7.5%) profile of Spectrin_N_ (2.0 µg, lane 1) and spectrin_VL_ (2.0 µg, lane 2), purified from RBC_N_ and RBC_VL_ as described by Ungewickell et al [31]. Purified spectrin_VL_ was further passed through an Achatinin-H-Sepharose 4B affinity column and 9-*O*-acetylated sialic acid containing spectrin_VL_ (2.0 µg, lane 3) was purified as described in Materials and Methods. Lane M shows molecular weight standards. ***B.*** *Presence of 9-O-AcSA as detected by Western blot analysis.* Equal amounts (2 µg) of purified spectrin_VL_ and spectrin_N_ were transferred onto nitrocellulose membrane after SDS-PAGE (8.5%). The blots were incubated overnight at 4°C with Achatinin-H and processed as described in Materials and Methods. **C.** Equal amount (2 µg) of purified spectrin_VL_ and spectrin_N_ were separated both on 5 and 7.5% SDS-PAGE under similar conditions. ***D.*** *Two dimensional (2D) gel electrophoresis of spectrin_VL_.* A representative 2D (pI range 4–7, 4–15% gradient) profile of purified spectrin (100 µg) from RBC_VL_ after staining with Coomassie is shown.

The identification of erythrocytic spectrin by mass spectrometry as *O*-acetylated sialoglycoprotein, prompted us to explore the status of total spectrin in RBC_VL_ (spectrin_VL_). Accordingly, spectrins were separately purified by the method as described by Ungewickell et al. [31]. The yield of spectrin_VL_ purified from ghost membrane (1.489±0.064 mg) of RBC_VL_ was 0.231±0.017 mg. Purified spectrin from RBC_N_ (spectrin_N_) demonstrated only two bands corresponding to α-, β-spectrin on SDS-PAGE (Fig. 2A, lane 1). In contrast, purified spectrin_VL_ exhibited an additional 60 kDa fragment along with α-, β-spectrin bands (Fig. 2A, lane 2). Purified spectrin_VL_ was further allowed to bind with Achatinin-H-Sepharose 4B. Achatinin-H bound spectrin_VL_ demonstrated three similar bands (Fig. 2A, lane 3). These observations confirmed the presence of 9-*O*-AcSA on α-, β-spectrin and 60 kDa band of RBC_VL_, which was indicative of alteration of spectrin in VL.

Western blot analysis of purified spectrin_VL_ separately by the method as described by Ungewickell et al [31] also showed reactivity with Achatinin-H reconfirming the identity of all three bands as spectrin containing 9-*O*-AcSA (Fig. 2B). In contrast, similar analysis of spectrin_N_ demonstrated the absence of all these three bands suggesting lack or undetectable 9-*O*-AcSA on RBC_N_.

SDS-PAGE analysis (5 and 7.5%) of spectrin_N_ and spectrin_VL_ demonstrated slight variation in their electrophoretic mobility (Fig. 2C) suggesting some changes in VL. Two dimensional gel electrophoresis of purified spectrin_VL_ reveals that individual spots corresponding to α-spectrin and β-spectrin and 60 kDa band have multiple isoelectric points (pI) (Fig. 2D) suggesting microheterogeneity of each spot possibly due to the differential sialylation of the same protein resulting into different pI.

### Identification of 60 kDa band of erythrocytic α-spectrin in RBC_VL_

**Figure 3:**
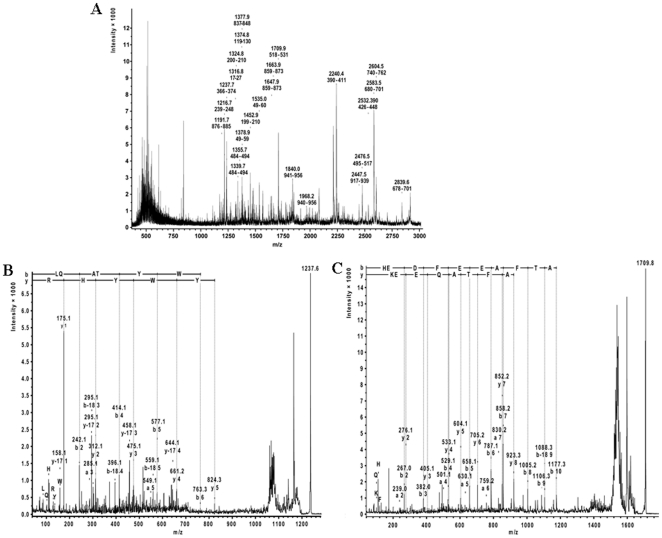
Identification of 60 kDa band. ***A.*** *The PMF spectra of tryptic fragments of 60 kDa glycoprotein.* PMF spectra of tryptic fragments of 60 kDa were identified as N-terminal fragment of α-spectrin by MALDI-TOF MS. Each fragment is denoted by their m/z values and sequence range within the 955 amino acids of human α-spectrin (marked with yellow in Fig. S1). ***B–C.*** *Confirmation of the sequence of the identified tryptic fragments by MALDI-TOF-TOF mass spectrometry.* The MS/MS spectrum was analyzed with database-dependent MASCOT as well as database-independent Sequit! software systems yielding the same results. Two representative PSD spectra of the MS/MS analysis of the fragment (B) LQATYWYHR (m/z = 1237.6) and (C) HEDFEEAFTAQEEK (m/z = 1237.6) of α-spectrin and SGP-60. The N and C terminal fragment ions are denoted according to standard nomenclature and immonium ions displayed in single amino acid code.

The 60 kDa band was identified as a fragment of *α*-spectrin (gi: 119573202) by mass-spectrometric PMF analysis (Fig. 3A) as well as sequence determination of fragments from PSD spectra. Representative MS/MS spectra of tryptic fragments of m/z = 1237.6 (Fig. 3B) and m/z = 1709.8 (Fig. 3C) are shown. The 24 detected and annotated tryptic fragments matched the *N*-terminal section of *α*-spectrin with sequence coverage of 18.4% for the entire spectrin sequence and of 36.1% for the *N*-terminal exons (Table 1).

**Table 1:** The 24 tryptic fragments of 60 kDa band determined by MALDI-TOF-MS analysis.

Mass [M+H]^+^	Sequence range	Deviation from theoretical mass	Missed cleavage	Sequence
1316.8079	17–27	0.14	0	VLETAEEIQER
1378.8979	49–59	0.19	0	LEDSYHLQVFK
1534.9679	49–60	0.17	1	LEDSYHLQVFKR
1374.8279	119–130	0.21	0	FTMGHSAHEETK
1452.9379	199–210	0.17	1	KFEDFQVELVAK
1324.8279	200–210	0.15	0	FEDFQVELVAK
1216.6979	239–248	0.13	0	QNEVNAAWER
1237.7579	366–374	0.14	0	LQATYWYHR
2240.4279	390–411	0.28	0	TAAINADELPTDVAGGEVLLDR
2532.3879	426–448	0.27	0	FQSADETGQDLVNANHEASDEVR
1339.7179	484–494	0.16	0	DSEQVDSWMSR
1355.7279	484–494	0.17	0	DSEQVDSWMox.SR
2476.5179	495–517	0.29	0	QEAFLENEDLGNSLGSAEALLQK
1709.9579	518–531	0.23	0	HEDFEEAFTAQEEK
2839.5879	678–701	0.21	1	QKGTLHEANQQLQFENNAEDLQR
2583.4879	680–701	0.27	0	GTQLHEANQQLQFENNAEDLQR
2604.5279	740–762	0.28	0	QDQVDILTDLAAYFEEIGHPDSK
1377.8679	837–848	0.12	0	VILENIASHEPR
1647.9479	859–873	0.20	0	MVEEGHFAAEDVASR
1663.9579	859–873	0.22	0	Mox.VEEGHFAAEDVASR
1191.7279	876–885	0.14	0	SLNQNMESLR
2447.4879	917–939	0.29	1	EKEPIVDNTNYGADEEAAGALLK
1968.2079	940–956	0.26	1	KHEAFLLDLNSFGDSMox.K
1840.0479	941–956	0.18	0	HEAFLLDLNSFGDSMox.K

The tryptic fragments matched the N-terminal portion of human erythrocytic α spectrin as compared to the protein sequences of the NCBI sequence database. The identification was confirmed by complete *de novo* sequencing of two fragments (shown in Figure 3 B–C). Mass [M+H]^+^ denotes the mono-isotopic masses of the fragment ions; sequence range refers to the alignment of the sequence of the denoted fragments with the α-spectrin reference sequence (gi: 119573202); deviation from theoretical mass is the mass difference between the measured mass and the mass calculated from the corresponding database sequence; missed cleavage refers to the missed trypsin cleavage sites in the identified fragment; sequence is the fragment sequence in one-letter code, Mox is oxidized methionine.

### Erythrocytic spectrin_N_ and spectrin_VL_ are glycosylated

**Figure 4:**
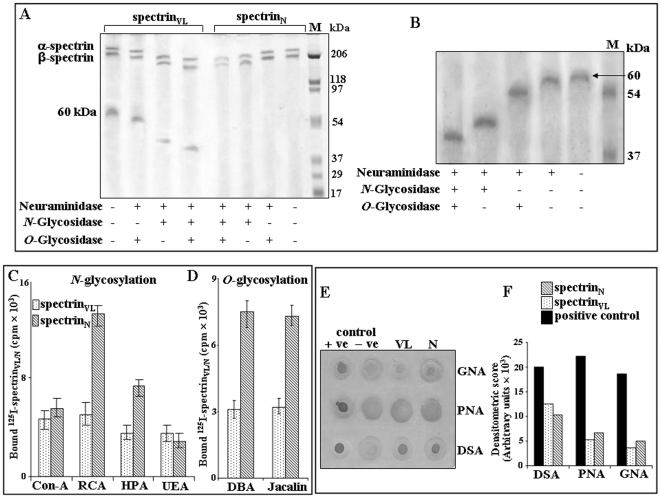
Demonstration of *N*-and *O*-glycosylation. ***A.*** *Demonstration of N-and O-glycosylation of spectrin by enzyme deglycosylation.* Equal amount (5 µg) of purified spectrin_VL_ and spectrin_N_ was treated with neuraminidase from *Arthrobacter ureafaciens* to remove the terminal sialic acids and subsequently desialylated spectrin_VL_ and spectrin_N_ was incubated separately with *N*-glycosidase F, *O*-glycosidase or a combination of *N*- and *O*-glycosidase as indicated. Spectrin_VL/N_ before and after the respective enzyme treatments were analyzed by SDS-PAGE as described in Materials and Methods. ***B.*** *Demonstration of sialylation, N- and O-glycosylation in 60 kDa fragment.* Gel-eluted purified 60 kDa fragment (1.0 µg) was initially desialylated with *Arthrobacter ureafaciens* neuraminidase overnight at 37°C. Subsequently the desialylated 60 kDa fragment was treated separately with *N*-glycosidase F, *O*-glycosidase F or a combination of both and analyzed by SDS-PAGE (7.5%) along with the untreated protein as described in Materials and Methods. Gel was stained with silver staining method. Lane M shows molecular weight standards. ***C–D.*** *Demonstration of N- and O-glycosylation by lectin binding with ^125^I-spectrin_VL/N_.* Fixed concentrations of ^125^I-spectrin_VL/N_ were processed separately to demonstrate their binding with several Sepharose/agarose bound ConA, RCA, HPA, UEA, DBA and Jacalin lectins (25 µl bead volume) having different sugar-linkage specificity as described in Materials and Methods. ***E–F.*** *Demonstration of N- and O-glycosylation by lectin binding with DIG-glycan.* **E.** Equal amount (2.0 µg) of spectrin_VL_ and spectrin_N_ was dot blotted on NC-paper and analyzed by DIG-glycan and differentiation kit using several lectins (GNA, PNA, DSA) following manufacturer's protocol. **F.** Representative bar graph of densitometric scores of corresponding spots.

The identification of sialic acids in spectrin_VL_ prompted us to explore the status of glycosylation of spectrin in RBC_N_. The presence of comparable *N*- and *O*-glycosylation was demonstrated by a shift in the respective protein bands corresponding to α- and β-spectrin following enzymatic deglycosylation of neuraminidase treated purified spectrin_N_ and spectrin_VL_ due to their reduced molecular mass (Fig. 4A).

In parallel, neuraminidase treated 60 kDa fragment was also exposed to *N*- and *O*-glycosidase F which evidenced a shift of the band corresponding to a reduction of molecular weight by ∼13.12 kDa and ∼3.12 kDa indicating presence of both *N*- and *O*-glycosydic bonds (Fig. 4B). The 60 kDa fragment also demonstrated a shift of ∼1.25 kDa after desialylation. Hence, deglycosylation accounted for about ∼16.24 kDa of the total mass of 60 kDa.

The existence of both *N*- and *O*-glycosylation was further confirmed by binding with Sepharose/agarose bound specific lectins using iodinated spectrin_VL_ and spectrin_N_ (Fig. 4C–D). The binding of ^125^I-spectrin_N_ with immobilized Concanavalin A (ConA), *Ricinus communis agglutinin* (RCA), *Helix pomatia agglutinin* (HPA) and *Ulex europaeus agglutinin* (UEA) clearly suggested the existence of *N*-glycosylation. Similarly the binding of *Dolichos biflorus agglutinin* (DBA) and Jacalin reflected the presence of *O*-glycosylation in ^125^I-spectrin_N_. These lectins also showed affinity towards the *N*- and *O*-glycosylated sugars present in ^125^I-spectrin_VL_.

Immobilized ConA and UEA showed comparable binding with ^125^I-spectrin_N_ and ^125^I-spectrin_VL_ suggesting equivalent glycosylation levels having α-Man (mannose), α-Glc (glucose) and α-L-Fuc (fucose) (Fig. 4C). However immobilized RCA, HPA, DBA and Jacalin showed higher binding towards ^125^I-spectrin_N_ as compared to ^125^I-spectrin_VL_ suggesting the presence of more terminal β-D-Gal (galactose) (GalNAc, β-Gal), α/β-D-GalNAc, α-GalNAc and β1-3GalNAc sugars in spectrin_N_ than spectrin_VL_.

The occurrence of Man α (1–3), (1–6) and (1–2) Man, Gal β (1–3) GalNAc and Gal β (1–4) GlcNAc (N-acetylglucosamine) in spectrin_N_ and spectrin_VL_ was further demonstrated based on the comparable binding with *Galanthus nivalis* agglutinin (GNA), peanut agglutinin (PNA) and *Datura stramonium* agglutinin (DSA) respectively using DIG-glycan differentiation kit (Fig. 4E–F).

### Erythrocytic spectrin_VL_ is highly sialylated

**Figure 5:**
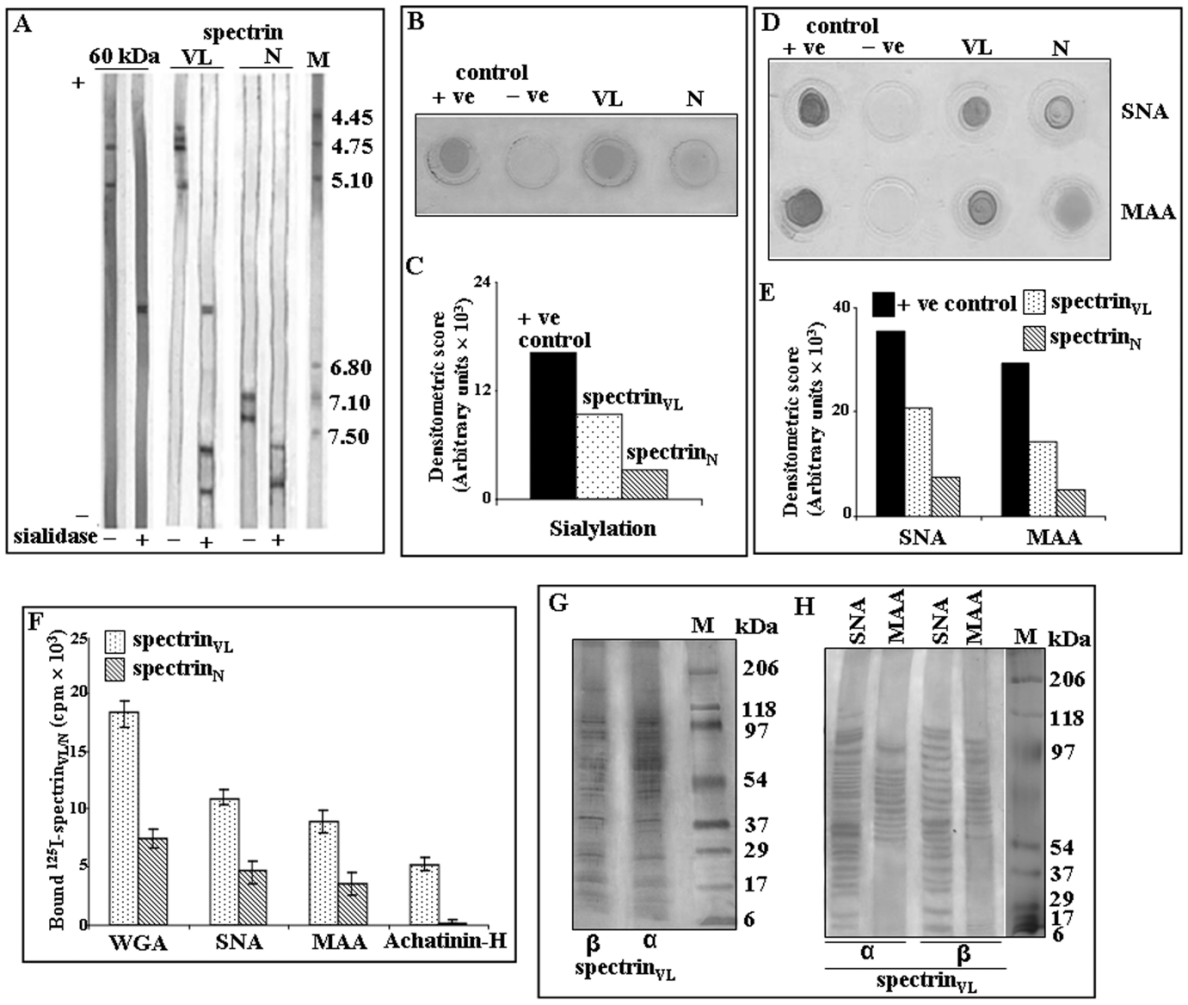
Presence of Neu5Ac and Neu5,9Ac_2_ in spectrin_VL_ by biochemical methods. ***A.*** *Enhanced sialylation demonstrated by IEF.* Equal amounts (3.0 µg) of purified spectrin_VL_, 60 kDa band and spectrin_N_ before and after removal of sialic acids were analyzed by IEF within a pH gradient of 3–10 and the respective bands visualized by silver staining. Lane M shows the pI markers. B–C. *Enhanced sialylation in spectrin_VL_.* Equal amount (1.0 µg) of purified spectrin_VL_ and spectrin_N_ was analyzed by using DIG-glycan detection kits and total sialylation was compared based on the densitometric scores of spots (B). Representative bar graph of densitometric scores of corresponding spots (C). *D–E. Detection of linkage-specific terminal sialic acids in spectrin_VL_.* Equal amount (2.0 µg) of spectrin_VL_ and spectrin_N_ was dot blotted on NC-paper and analyzed by DIG glycan and differentiation kit using SNA and MAA lectins following manufacturer's protocol (D). Densitometric scores of corresponding spots are shown as bar graph (E). *F. Binding of ^125^I-spectrin_VL/N_ with several sialic acid binding lectins.* To demonstrate the presence or absence of terminal sialic acids, a fixed concentrations of ^125^I-spectrin_VL/N_ were analyzed by binding with Sepharose/agarose bound WGA, SNA, MAA, Achatinin-H (25 µl bead volume) having specificity towards linkage specific sialic acids as described in materials and methods. Bound radioactivity of ^125^I-spectrin_VL/N_ was measured by Gamma-counter and represented as bar graphs. ***G–H.*** *Detection of sialylated tryptic fragments in spectrin_VL_.* The α and β subunits of purified spectrin_VL_ were digested separately by restricted amount of trypsin. Such controlled digested and extracted tryptic fragments were dried and redissolved and an aliquot was separated in SDS-PAGE (7.5%–15% gradient) (G). Subsequently the presence of sialic acids on resulting tryptic fragments was analyzed by binding with SNA-agarose and MAA-agarose separately and followed by electrophoresis on SDS-PAGE (7.5%–15% gradient) (H) as described in Materials and Methods. Lane M shows the molecular weight standerds.

Isoelectric focusing (IEF) of spectrin_VL_ demonstrated four distinct bands within a pI range of 4.6–5.21 (Fig. 5A), which showed a considerable shift of their pI to a range of 6.25–7.95 after neuraminidase treatment indicating the presence of sialic acids. Furthermore the homogeneous shifts of the individual bands demonstrated the homogeneity of the proteins. In contrast shift in pI of spectrin_N_ before and after neuraminidase treatment was less marked suggesting lower degree of sialylation. IEF of 60 kDa fragment showed two distinct bands, which demonstrated a shift in their pI after desialylation. This indicated that 60 kDa fragment comprised of two fragments of similar mass having sialic acids. Lane M shows the pI markers.

### Enhanced presence of SA in spectrin_VL_ as detected by biochemical and glycoanalytical methods

The presence of total sialic acid was demonstrated in spectrin_VL/N_ using DIG-glycan detection kit (Fig. 5B–C). Spectrin_VL_ showed enhanced (3-fold) sialylation as compared to spectrin_N_. The variable expression of linkage-specific sialic acids was demonstrated (Fig. 5D–E). Spectrin_VL_ showed ∼2.7–2.8 fold enhanced binding with *Sambucus nigra* agglutinin (SNA) and *Maackia amurensis* agglutinin (MAA) compared to spectrin_N_.

Sialylation were further demonstrated by binding with iodinated spectrin_VL_ and spectrin_N_ with Sepharose/agarose bound Wheat germ agglutinin (WGA), SNA and MAA. Spectrin_VL_ showed significantly higher binding (∼2.4 fold) with all three lectins than spectrin_N_ (Fig. 5F). Achatinin-H also showed higher binding with spectrin_VL_ whereas negligible with spectrin_N_ (Fig. 5F).

With an attempt to search for the presence of sialylated tryptic fragments, the α- and β-subunits of spectrin_VL_ were partially digested with trypsin separately. This controlled digestion yielded many fragments though we might have missed many smaller fractions (Fig. 5G). Theses fragments were allowed to bind with SNA and MAA agarose separately. SNA and MAA bound fragments were analysed on SDS-PAGE. Approximately 25 fragments of α-spectrin_VL_ showed α2,6 linked and 18 of them had α2,3 linked terminal sialic acid. β-spectrin_VL_ showed comparatively less number of α2,6 and α2,3 linked terminal sialic acids containing fragments (Fig. 5H).

**Figure 6:**
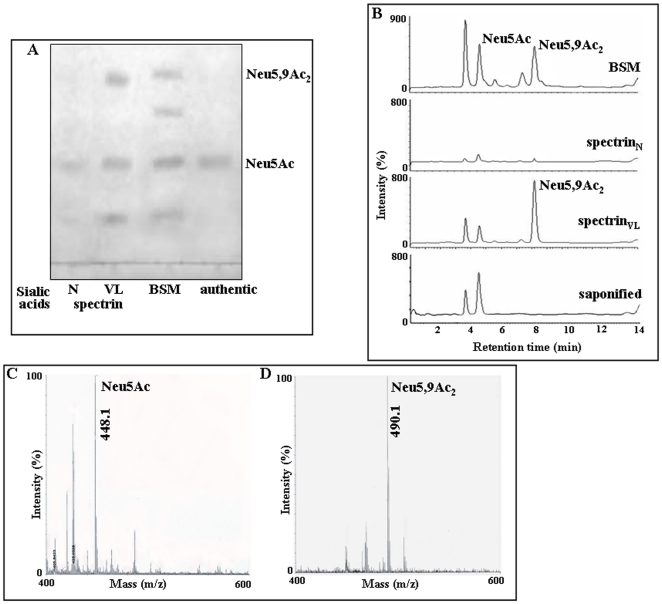
Presence of Neu5Ac and Neu5,9Ac_2_ in spectrin_VL_ by analytical methods. ***A.*** *Thin layer chromatography (TLC).* Glycosidically bound sialic acids of spectrin_VL_ were subjected to acid hydrolysis, purified, separated on a TLC plate and detected by staining with orcinol/HCl spray reagent and baking at 180°C. Similarly processed free sialic acids released from BSM served as standard. Additionally, commercially available Neu5Ac was used as references. For comparison liberated sialic acids from purified spectrin_N_ were similarly analyzed. ***B.*** *Enhanced presence of Neu5Ac and Neu5,9Ac_2_ in spectrin_VL_ as determined by fluorimetric HPLC.* Glycosidically bound sialic acids released from spectrin_VL_ by acid hydrolysis were derivatized with DMB and analyzed by fluorimetric HPLC before and after saponification as described in Materials and Methods. A representative chromatogram of the spectrin_VL_ and spectrin_N_ derived sialic acids showed the presence of fluorescent derivatives of free sialic acids. In parallel sialic acids of BSM similarly analyzed under identical conditions served as standard. ***C–D.*** *Identification of sialic acids by MALDI-TOF MS.* Fractions corresponding to peaks of Neu5Ac (C) and Neu5,9Ac_2_ (D) were collected after fluorimetric HPLC, spotted and analyzed by MALDI-TOF MS using DHBA matrix as described in Materials and Methods. Positive ion mode was used for mass-spectrometric analysis with 1000 laser shots per spot.

Glycosidically bound sialic acids (SA) liberated from spectrin_VL_ when separated on a TLC (thin layer chromatography) plate demonstrated the presence of Neu5Ac (N-acetyl neuraminic acid) and Neu5,9Ac_2_ (5,9-diacetyl neuraminic acid) as compared with standard Neu5Ac and free SA purified from bovine submandibular mucin (BSM) (Fig. 6A). The presence of these derivatives was also demonstrated in the chromatogram of the liberated SA from spectrin_VL_ by fluorimetric high-performance liquid chromatography (HPLC) (Fig. 6B). The Neu5,9Ac_2_ peak of spectrin_VL_ completely disappeared on saponification. In contrast, spectrin_N_ showed undetectable Neu5,9Ac_2_ suggesting disease-associated modification. BSM-derived SA showing ∼40% Neu5,9Ac_2_ was used as standard. Each fraction corresponding to Neu5Ac and Neu5,9Ac_2_ of spectrin_VL_ was collected after fluorimetric-HPLC and was subsequently confirmed by MALDI-TOF-MS which yielded their expected molecular ion signals having m/z at 448.1 (Fig. 6C) and 490.1 (Fig. 6D) respectively.

### Molecular modelling of glycosylated residues

In α-spectrin, four potential *N*-glycosylation sites were identified all of which contains the consensus sequence, Asn-Xaa-Ser/Thr (Table 2). However, only one potential *O*-glycosylation site was found at Thr-817 position with a score above the threshold value (0.35). However, in β-spectrin, two potential *N*-glycosylation sites were found and no potential *O*-glycosylation site (Table 2).

**Table 2:** Potential glycosylation sites and solvent accessibility values of spectrin_N_.

Spectrin_N_	Glycosylation sites^a^	Accessible surface area [Å^2^]
α-spectrin	Asn-Lys-Thr [633–635]	16.94
	Asn-Val-Thr [657–659]	75.37
	Asn-Thr-Ser [1625–1627]	93.70
	Asn-Leu-Ser [2077–2079]	52.24
	Thr-817	142.89
β-spectrin	Asn-Val-Thr [194–196]	45.05
	Asn-Phe-Thr [197–199]	21.97

a *N*-glycosylation sites (Asn) are shown as the consensus sequence of three amino acids and *O*-glycosylation site (Thr) is shown as the single amino acid. Sequence numbering is done according to the human alpha spectrin , erythrocytic 1, isoform CRA_b (gi: 119573202, taken from NCBI protein sequence database).

Structural verification of the predicted models of the modules containing *N*- and *O*- linked glycosylation sites revealed that the backbone conformations were satisfactory as the allowed phi-psi combinations were above 90% in the allowed region of Ramachandran's plot. Verify 3-D results showed that the models have an average 85% of the residues with 3D-1D score greater than 0.2 which indicates good quality 3D structural parameters. From ERRAT analysis it was observed that most of the models have an overall quality factor greater than 90% indicating good structural quality.

**Figure 7:**
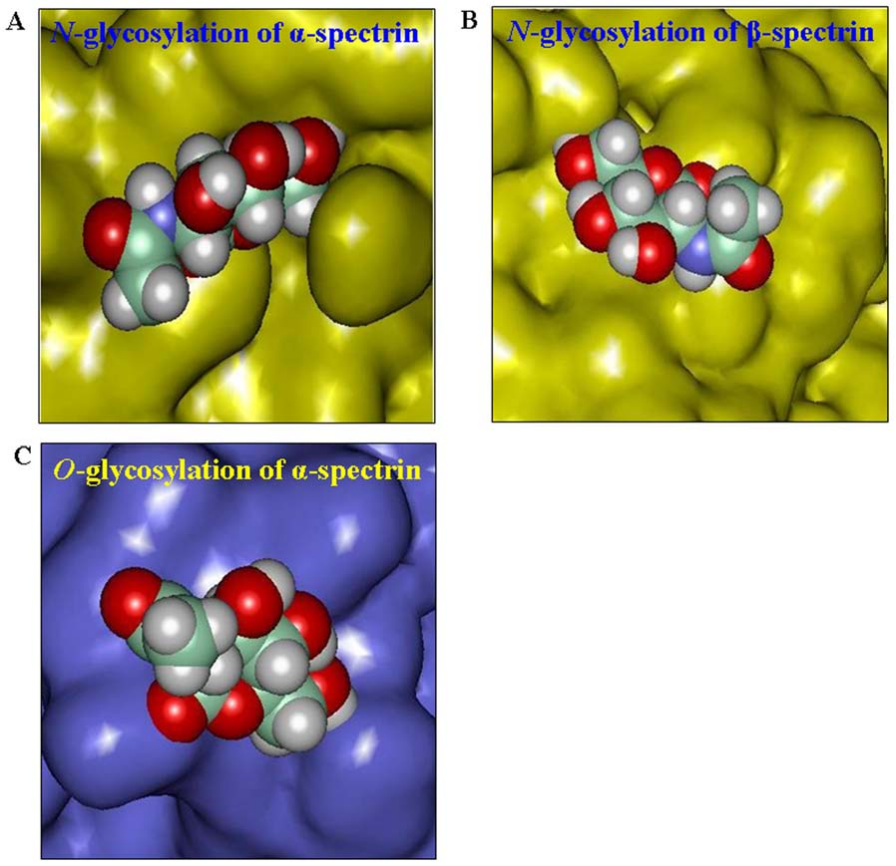
Space filling structural representation of GlcNAc in spectrin_N_. Sugar moiety are colored by atoms (C = green, O = red, N = blue and H = white). The protein model is represented as conolly surface. **A.** *N*- glycosylation of α-spectrin is shown in yellow color at position Asn-1625. **B.** *N*- glycosylation of β-spectrin is shown in yellow color at position Asn-194. **C.** *O*- glycosylation of α-spectrin is shown in blue color at position Thr-817.

Solvent accessible surface areas was calculated by ACCESS and the probable glycosylation sites shows that the residues are exposed enough needed for glycosylation at the sites (Table 2). Further, modelling of a representative sugar (β-GlcNAc) into one each of *N*- and *O*- glycosylation sites showed that the sugar moiety can go into the available space around the amino acid residues (Fig. 7).

### Spectrin_N_ and spectrin_VL_ showed slight variations in their secondary structures

**Figure 8:**
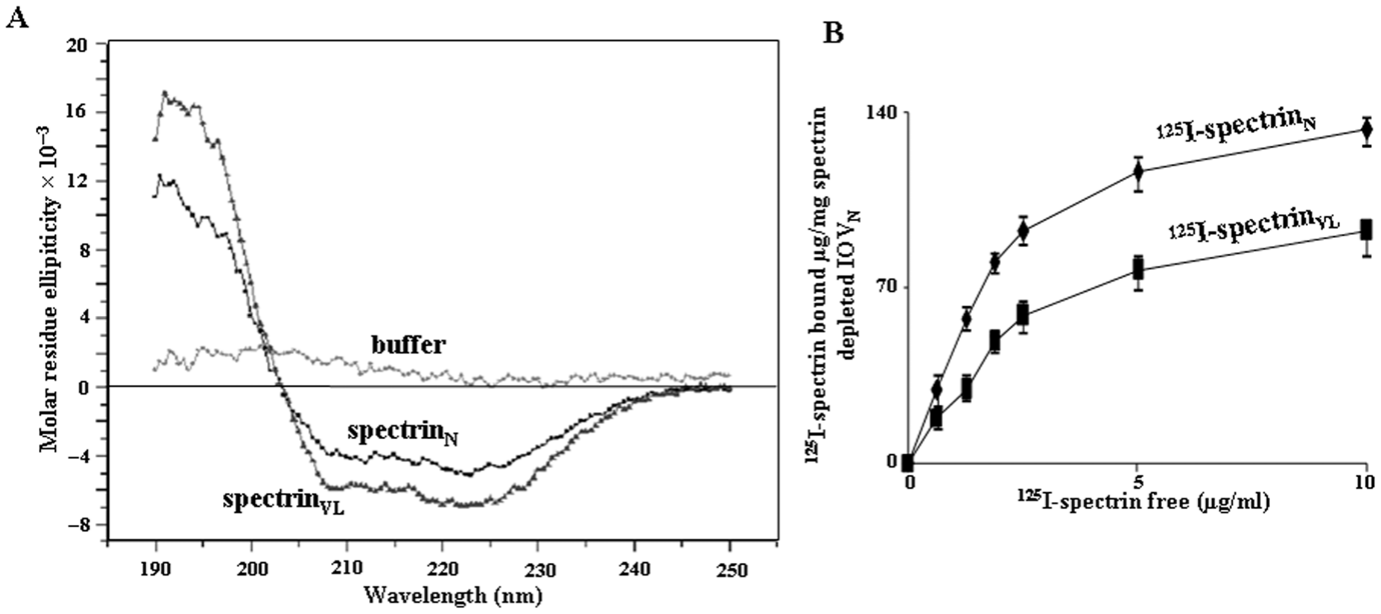
Physicochemical study of structural modification of spectrin_VL_. ***A.*** *CD-spectra.* Far-UV CD spectra of spectrin_VL_ and spectrin_N_ in phosphate buffer (20 mM, pH 7.0) indicating the molar residue ellipticity as a function of wavelength along with the buffer only. ***B.*** *Binding of ^125^I-spectrin to spectrin-depleted IOV_N_.* Various concentrations of ^125^I-spectrin_VL/N_ were incubated with a constant amount of spectrin-depleted-IOV_N_ followed by determination of specific binding as described in Materials and Methods.

The CD (Circular Dichroism) spectra of spectrin_N_ in far-UV (ultra violet) region showed that protein contains 51.71% of α-helix, 9.17% of β-sheet and 39.12% of random coil (Fig. 8A). The similar trend was observed in the secondary structure prediction using GOR 4 [32]. The sequences of α- and β-spectrin_N_ was taken as weighted averages; the values of α-helix, β-sheet and random coil are 71.61%, 4.98% and 23.28% respectively. In parallel, the values for α-helix, β-sheet and random coil are 77.9%, 3.03% and 18.71% respectively as predicted from the modelled structures using MODELYN [33].

However, spectrin_VL_ demonstrated a slight increase of α-helicity (63.05%) and a minimal decrease of β-sheet (5.68%) structure suggesting higher degree of sialylation possibly playing a role for such minute changes in its secondary structure.

### Binding of ^125^I-spectrin_N_ and ^125^I-spectrin_VL_ to spectrin-depleted inside-out membrane vesicles (IOV)

In order to further demonstrate the modified structure of spectrin_VL_ in comparison to spectrin_N_ we have compared the binding status of iodinated spectrin_VL_ and spectrin_N_ with spectrin-depleted IOV from normal RBC-ghost (IOV_N_). The binding of ^125^I-spectrin_VL_ to spectrin-depleted IOV_N_ increases with increasing amount of ^125^I-spectrin_VL_ (Fig. 8B). In contrast, under identical condition, ^125^I-spectrin_N_ showed much higher binding towards spectrin-depleted IOV_N_. Such differences in binding signifies that minute structural modifications due to enhanced sialylation in spectrin_VL_ possibly make it less available for interacting with other associated proteins in the spectrin-depleted IOV_N_ of RBC_N_.

## Discussion

VL is often complicated by anemia. The exclusive presence of 9-*O*-AcSGPs on erythrocytes of active VL has been correlated to RBC hemolysis [18], [20]. The functional attributes of erythrocytes are determined by the structural integrity of the membrane, which is often described in terms of alterations in the membrane characteristics like osmotic fragility, fluidity and hydrophobicity. Any kind of perturbation in the milieu of the erythrocytes like oxidative changes or ligand specific interaction culminates in changes in the membrane characters generally associated with pathological conditions [25]. Therefore, we considered it worthwhile to unravel the molecular determinants and implications of 9-*O*-AcSGPs on RBC_VL_.

The major observation of this study is the demonstration of glycosylation in normal spectrin purified from RBC_N_, presence of higher degree of sialylation in spectrin purified from RBC_VL_ and fragmentation of spectrin_VL_ as a 60 kDa 9-*O*-AcSGP. Therefore, it may be envisaged that enhanced sialylation of spectrin_VL_ is possibly responsible for the generation of this fragmented *O*-acetylated sialic acid-containing spectrin_VL_. Altered binding of highly sialylated spectrin_VL_ with spectrin-depleted inside-out membrane vesicles of RBC_N_ possibly suggested functional abnormality. Membrane characteristics of RBC_VL_ were observed by enhanced hydrophobicity, fragility, fluidity as compared to RBC_N_ hinting towards membrane damage [25].

We have purified eight distinct 9-*O*-AcSGPs from RBC_VL_ using Achatinin-H as an affinity matrix indicating linkage specific terminal 9-*O*-AcSA in these sialoglycoproteins. Distinct multiple spots of individual 9-*O*-AcSGP suggested microheterogeneity possibly due to differential sialylation. As 9-*O*-AcSGPs are exclusively present on RBC_VL_, their identification through mass spectrometry was necessary to assess their possible implication in the disease pathology.

The analysis of two high molecular weight 9-*O*-AcSGPs by MALDI-TOF-MS evidenced a match with the NCBI entry of human erythrocytic α and β-spectrin with sequence coverage of 33.6% and 22.7% respectively. The amino acid sequences of tryptic fragments deduced from MS analysis confirmed the identification. Sequencing of two tryptic fragments and database-dependent Mascot as well as database-independent Sequit analyses made the identification unambiguous.

The cytoskeleton beneath the lipid bilayer of the membrane of RBC comprises of several proteins interconnected with each other providing stability and integrity to the membrane structure. Spectrin exists as a heterotetramer consisting of two subunits each of α-(280 kDa) and β-(246 kDa) spectrin oriented in an anti-parallel arrangement. The presence of both *N*- and *O*-glycosylation was indicated by shifts in molecular mass after the respective glycosidase treatments of neuraminidase-treated spectrin_VL_ and spectrin_N_. Binding with several lectins specific for *N*- and *O*-glycosylation also supported the presence of such glycosylation in spectrin_VL_ and spectrin_N_.

Molecular modelling studies also supported both *N*- & *O*- glycosylations of α-specrin. However, only *N*-glycosylation was found in β-spectrin. Modelling the sugar moiety to the predicted glycosylation sites suggested that glycans could fit into these sites without any steric clashes, thus signifying the probability of glycosylation of spectrin.

Cell surface sialic acids have been widely associated with different pathological conditions. Enhanced presence of sialic acids in spectrin_VL_ has been convincingly exhibited by lectin binding, which was further confirmed by TLC, fluorimetric-HPLC and MALDI-TOF-MS. Demonstration of pI of spectrin_VL_ in acidic region and a huge shift of pI after neuraminidase treatment established enhanced sialylation compared to spectrin_N_. More importantly, exclusive presence of Neu5,9Ac_2_ in spectrin_VL_ suggested disease-associated enhanced sialylation in VL. Enhanced sialylation in spectrin_VL_ compared to spectrin_N_ possibly causes structural modification of spectrin_VL_. Such structural changes were perhaps the basis for the reduced capacity of spectrin_VL_ to complex with other associated cytoskeletal proteins in normal environment as demonstrated by its less binding with spectrin-depleted IOV_N_.

Interestingly, purified 60 kDa fragment demonstrated the presence of two distinct bands in IEF and each of the bands depicted a distinct shift in their pI after removal of SA showing the presence of two sialylated proteins of similar molecular mass. In contrast spectrin_N_ having comparable glycosylation, showed complete absence of such fragmentation, which suggested that alteration of spectrin mainly enhanced sialylation may be associated with VL pathology.

The cleaved 60 kDa fragment which belongs to α-spectrin contains two potential *N*-linked glycosylation sites, at Asn-633 & Asn-657 and one *O*-linked glycosylation site at position Thr-817 with sufficient surface accessibility. The remaining portion of α-spectrin although contains two potential *N*-linked glycosylation sites, but no *O*-linked glycosylation site was found. On the other hand, we could not identify any potential *O*-linked glycosylation site in the β-spectrin. Therefore, it may be envisaged that the exclusive presence of *O*-linked glycosylation site in the *N*-terminal region of α-spectrin with high surface accessibility tends to have higher sialylation for interaction with each other. All these factors combined may play important role in the cleavage of α-spectrin to 60 kDa fragment in VL.

Production of erythrocytes requires synthesis of red cell proteins specially cytoskeleton proteins. During terminal differentiation of erythroid progenitor cells in culture it retains the key components of the endoplasmic reticulum protein translocation, glycosylation, and protein folding machinery, chaperones, calreticulin and Hsp90 for red cell glycoprotein biosynthesis [34].

Non-enzymatic glycation and oxidation of spectrin were reported under several physiological conditions [3]. Such non-enzymatic changes are a result of glyco-oxidation, where the oxidative stress within, surpasses the antioxidant defense system of the cell [35]. The associated biochemical alterations affect the structure, aggregation and integrity of the membrane and membrane-associated proteins. Such changes have been witnessed in erythrocytic spectrin of subjects suffering from long-term diabetes mellitus. Here the elevated glucose concentration increases oxidation and advanced glycation end product formation of structural and membrane proteins of erythrocyte [36]. The demonstration of spectrin_VL_ with enhanced sialylation in VL patients raised questions regarding the basis of these modifications. Therefore, it may be envisaged that oxidative modification of spectrin affects membrane morphology of the erythrocytes. Enhanced fragility, membrane fluidity and hydrophobicity of RBC_VL_ as compared to RBC_N_ were demonstrated earlier [25]. Hence, the evidence of altered spectrin reported here may provide an explanation for the known-impaired stability of erythrocytes in VL.

The presence of elevated levels of serum sialic acids in cardiovascular diseases and their relation to evaluated myocardial cell damage have been documented and it has been suggested that either the shedding or secretion of cell membrane sialic acids determines their accumulation in serum [37], [38]. Furthermore, the importance of elevated serum sialic acids and soluble sialyltransferases in the diagnosis of Down-syndrome affected pregnancy and oral cavity cancer has been documented [39], [40]. The presence of sialyltransferases in human serum may provide a possible way of changes in serum proteins with terminal α2-6 sialic acid [40]. Bulai et al. have characterized a transport system and demonstrated the uptake of free sialic acids into human erythrocytes [41]. Therefore, free sialic acids could be transported across the membrane into RBC through a sialic acids transport system. Interestingly, the presence of enhanced sialic acids in the serum of VL patients probably hinted towards a possible mechanism of transport of these free sialic acids under the influence of sialyltransferases. Hence presence of sialic acids in VL serum could essentially serve as a source for the erythrocyte sialic acids, which could use a transport system for their entry. The uptake of sialic acids was monitored by measuring free sialic acids and ManNAc produced by cytosolic sialate pyruvate-lyase in human erythrocytes that indicated the presence of a sialic acid transport system [42]. Furthermore, in VL, peripheral hematopoietic cells have increased sialic acids [26] that could be shaded in the serum and be transported across the erythrocyte membrane. The presence of serum sialyltransferases in VL patients and testing this hypothesis as well as the elucidation of the mechanisms of enhanced sialylation of spectrin in RBC_VL_ demands extensive studies and will be a subject of future investigations.

Taken together the current study provides evidence for the first time not only for glycosylation of spectrin_N_ but also enhanced sialylation in diseased condition i.e. spectrin in RBC_VL_. Additionally, we have demonstrated fragmented spectrin_VL_ which could be triggered by such enhanced sialylation. Therefore, we hypothesize that the higher sialylation along with exclusive presence of 9-*O*-AcSA on RBC_VL_ may in turn, cleaves spectrin, ultimately resulting in destabilization and functional inability of the RBC. From this entire study, we contend that these 9-*O*-AcSGPs trigger membrane damage and may serve as an important factor leading to anemia-associated with VL. Hence the study successfully dissects one of the causal mechanisms leading to anemia, a common manifestation in VL.

## Materials and Methods

### Clinical samples

Blood sample of clinically confirmed active VL patients (n = 30; 21 males, 9 females, median age: 30 years) based on microscopic demonstration of *Leishmania sp.* amastigotes in splenic aspirates were collected from School of Tropical Medicine, Kolkata and immediately processed for the separation of RBC_VL_ at Indian Institute of Chemical Biology. The diagnosis was validated by two in-house techniques, in which the increased presence of linkage-specific 9-*O*-AcSGPs was quantified by erythrocyte binding assay [17] and anti-9-*O*-AcSGPs antibodies were detected by enzyme-linked immunosorbent assay (ELISA) using BSM known to contain a high percentage of 9-*O*-AcSAs, as coating antigen [30], [23]. The hematological parameters evidenced anemia in these patients and ruled out any other blood cell disorder (Table 3). Existence of high level of sialic acid in VL serum was observed.

**Table 3:** Diagnostic features of patients with active visceral leishmaniasis (VL).

Parameters	Patient_VL_ (n = 30)	Normal (n = 30)
**Age (yr)**	20–40	20–40
**Weight (Kg)**	32–40	50–60
**Duration of illness (mo)**	4–6	Not applicable
**RBC count**	1.0–2.5×10^6^/µl	4.0–6×10^6^/µl
**Leukocyte count (/mm^3^)**	3–4×10^3^	5–10×10^3^
**Hemoglobin concn. (g/dl)**	4–5	10–12
**Reticulocyte count (%)**	4–5	1–3
**Spleen size (cm)**	7–10	Not palpable
**Splenic aspirate score** a	3.5–4.2	Negative
**RBC-ELISA**	0.95–1.38	0.19–0.24
**9-** ***O*** **-AcSGP^+^RBC** b	85–90%	0.08–0.12%
**BSM-ELISA** c	0.85–1.2	0.19–0.24
**Parasite ELISA** d	1.1–1.8	0.21–0.28
**Serum sialic acid content (mg/dL)** e	77.05±3.6	57.42±3.49

a 4, >1 to 10 parasites per field.

b Determined by flow cytometry using FITC-Achatinin-H [18].

c Anti-9-*O*-AcSGP antibody was detected by using BSM as coating antigen as described elsewhere [30].

d Parasite specific antibody was detected by using parasite lysate as coating antigen as described elsewhere [16].

e Sialic acid content in serum was estimated by thiobarbituric acid method [54].

Peripheral blood from normal human donors from endemic (n = 15) and non-endemic areas (n = 15) was processed similarly to obtain RBC_N_ for the study. The Institutional Human Ethical Committee had approved the study and samples were taken with the consent of the donors, patients, or in case of minors from their parents/guardians.

### Purification of 9-O-AcSGPs from RBC_VL_

RBC_VL_ (2×10^10^) after Ficoll-Hypaque (Amersham Pharmacia, Uppsala, Sweden) gradient separation comprising of 85–90% 9-*O*-AcSGP-positive cells were washed consecutively thrice with sodium chloride (NaCl, 0.15 M). Erythrocyte ghosts were prepared by sequential lyses of RBC_VL_ using 5.0 mM, 2.5 mM and 1.25 mM ice-cold phosphate buffer, pH 7.0. The ghost membranes were solubilized in solubilizing buffer containing Tris-HCl (0.05 M), 1% (w/v) detergent concentration (CHAPS∶BOG 1∶1), MgCl_2_ (1.0 mM), CaCl_2_ (1.0 mM), dithiothreitol (DTT, 0.2 mM), phenylmethyl sulfonyl fluoride (PMSF, 20 µg/L), protease inhibitor cocktail, pH 7.2, sonicated (three pulses, 10 sec each) in ice-mixture and incubated at 4°C for 1 hr [43]. After centrifugation at 8200×g, 4°C the supernatant was collected and dialyzed against Tris-HCl (0.05 M, pH 7.2) saline (TBS) containing 0.03 M Ca^2+^ (TBS-Ca^2+^), 0.01% (w/v) detergent (CHAPS∶ BOG 1∶1), sodium azide (0.02%). The dialyzed protein was processed for affinity chromatography and the protein content was quantified by Lowry method [44].

RBC_VL_ ghost membrane protein fraction (1.85 mg) was passed through Achatinin-H-Sepharose-4B affinity column (2.0 mg/ml) equilibrated with TBS-Ca^2+^ containing sodium azide (0.02%) at 4°C as described elsewhere [18]. After extensive washing, Achatinin-H-bound 9-*O*-AcSGPs were eluted at 25°C with TBS containing sodium citrate (0.04 M, pH 7.2), dialyzed against TBS at 4°C and stored at −70°C for future use. As the binding of Achatinin-H towards 7-*O*- and/or 8-*O*-AcSA cannot be ruled out, therefore, presence of such linkages in *O*-acetylated sialoglycoproteins are also possible.

### Protein/peptide mass spectrometry

The identification of the glycoprotein was done by mass spectrometry using Bruker-Daltonics MALDI-TOF mass spectrometer Reflex IV (Bruker Daltonics, Bremen, Germany). The samples were prepared by dried-droplet procedure using 2,5-dihydroxybenzoic acid (DHBA) as matrix. Calibration was done externally with a mixture of Angiotensin I, Angiotensin II, Substance P, Bombesine, ACTH clip 1–17 and ACTH clip 18–39. Subsequently, peptide samples were used for analysis. Tryptic fragments were generated by overnight in-gel digestion of two high molecular bands in ammonium hydrocarbonate (5 mM) using 10 ng trypsin (Promega, Mannheim, Germany) per sample. Sequence analysis of selected tryptic fragments was done with an Ultraflex III MALDI-TOF-TOF mass spectrometer (Bruker Daltonics). PSD spectra were acquired using default LIFT method for MS/MS spectra acquisition with manually adjusted laser energy accumulating data from 1500–2000 laser shots. Spectra annotation was done using the FlexAnalysis 3.0 (Bruker Daltonics) software. PMF analyses and MS/MS ion searches were done with MASCOT (Matrix Science Ltd., London, UK). Database searches through Mascot with PMF and MS/MS data were done with the BioTools 3.1 software (Bruker Daltonics). For database searches the following parameters were used. Taxonomy: Homo sapiens; database: NCBI; enzyme: trypsin; variable modifications: oxidation on methionine and one missed cleavages. Database searches for PMF spectra were done at the fragment mass tolerance ±0.3 Da. For the MS/MS searches mass tolerances for precursor was ±0.2 Da and 0.4 Da for fragment masses were used. The identification of the fragments and thereby of the protein was confirmed by database-independent *de novo* sequencing using the Sequit! Software [45].

### Purification of spectrin

The spectrin_N_ from RBC_N_ (1.25 mg total ghost membrane protein) was purified following the method of Ungewickell et al. with slight modifications [31]. Briefly, the ghosts were washed twice and resuspended in 3 volume of sodium phosphate (0.3 mM, pH 7.2) containing ethylene diamine tetraacetic acid (EDTA; 0.2 mM), (extraction buffer) and incubated for 20 min at 37°C. The fragmented ghosts were pelleted by centrifugation at 80000× g for 1 h at 2°C. Water-soluble proteins in the supernatant were immediately applied to a Sepharose 4B column (90×2 cm), equilibrated with Tris (25 mM), EDTA (5 mM), NaCl (0.1 M), pH 7.6 (Tris/EDTA/saline buffer) at 4°C. The column was eluted at 10 ml/h and 4-ml fractions were collected. Protein in the effluent was monitored by absorbance at 280 nm. Fractions containing purified spectrin dimer were pooled, concentrated and dialyzed overnight at 4°C against TBS-Ca^2+^ buffer containing sodium azide. In parallel, spectrin_VL_ was similarly purified from RBC_VL_. Additionally, the purified total spectrin_VL_ was further passed through an Achatinin-H-Sepharose 4B affinity column and 9-*O*-AcSA containing spectrin_VL_ was purified as described above.

### Electrophoresis

#### 2D Gel electrophoresis

Purified spectrin_VL_ was processed with 2D-clean up kit according to manufacturer's protocol (Bio-Rad, USA). The precipitated protein was dissolved in rehydration buffer containing urea (6 M), thiourea (2 M), CHAPS (2%), DTT (50 mM), carrier ampholyte cocktail (2%), EDTA (0.1 mM), bromophenol blue and quantitated by Quick Strat Bradford. Protein (100 µg/100 µl) was rehydrated on IEF strips (pI 4–7, 7 cm) for 8 hrs and ran in PROTEAN IEF cell. Strips were equilibrated consecutively in two steps of 30 min each in equilibration buffer containing urea (6 M), SDS (2%), Tris-HCl (0.375 M, pH 8.8), glycerol (20%), DTT (2%) followed by same buffer with iodoacetamide (2.5%). The second dimension was carried out on gradient (4–15%) SDS-PAGE and stained by Biosafe Coomassie brilliant blue [46].

Purified spectrin_N_, spectrin_VL_ or affinity-purified spectrin_VL_ and gel eluted 60 kDa fragment and were analysed by SDS-PAGE (5 and 7.5%) in a minigel apparatus (Bio-Rad, USA) [25] and the gels were stained. *N*- or *O*-linked glycosylation of spectrin_VL_, spectrin_N_ and 60 kDa fragment was demonstrated after deglycosylation with specific glycosidases using deglycosylation kit (Roche Applied Science, Mannheim, Germany) according to the manufacturer's protocol [26].

Western blot analysis [26] of spectrin_VL_ and spectrin_N_ was performed by semidry method at 15 V for 20 min. After blocking, the membranes were incubated with Achatinin-H (100 µg/ml) in the presence of Ca^2+^ (0.03 M). Subsequently, the Achatinin-H probed membrane was incubated with polyclonal rabbit anti-Achatinin-H antibodies (1∶400) at 4°C. Both the blots were developed using HRP-conjugated goat anti-rabbit IgG (1∶5000, Cell signaling) and detected using diaminobenzidine (Sigma, St. Louis, MO) as substrate.

To obtain pure 60 kDa protein, Coomassie-stained bands corresponding to 60 kDa were excised from the electrophoresis gels and the proteins eluted using an Electro-Eluter Model 422 (Bio Rad, USA). IEF of purified spectrin_VL_, gel-eluted 60 kDa fragment and spectrin_N_ was performed in capillary tubes within a pH range 3.0–10.0 using Mini-PROTEAN II tube cell apparatus (Bio-Rad, USA) and silver stained. The samples were desialylated overnight with *Arthrobacter ureafaciens* neuraminidase (0.2 mU/µg) at 37°C and processed similarly. The isoelectric points were determined from the pI of known proteins used as standards [26].

### Analysis of carbohydrates

#### DIG-glycan detection

Equal amounts (1.0 µg) of spectrin_VL_ and spectrin_N_ were dot blotted on nitrocellulose paper (NC-paper) and total sialic acid content was analyzed by using DIG-glycan detection kit (Roche Applied Science, Mannheim, Germany) following manufacturer's protocol [8]. Densitometric measurement of spots was done by using ImageQuantTL software (GE Healthcare).

#### DIG-glycan differentiation

The detection of terminal sugars on equal amount (2.0 µg, dot blotted on NC-paper) of spectrin_VL_ and spectrin_N_ was analyzed [26] by DIG-Glycan differentiation kit (Roche Applied Science, Mannheim, Germany) using several plant lectins SNA (specific for α2→6 linked Neu5Ac), MAA (specific for α2→3 linked Neu5Ac), GNA (specific for terminal Man (α1→3), (α1→6) and (α1→2) Man), PNA (specific for Gal (β1→3) GalNAc) and DSA (specific for Gal (β1→4) GlcNAc) according to the manufacturer's protocol. Densitometric measurement of spots was done as above.

#### Immobilized lectin binding assays to ^125^I-spectrin_VL/N_

To analyze the terminal sugar linkages, spectrin_VL_ and spectrin_N_ were separately iodinated with ^125^I (Bhabha Atomic Research Centre, Mumbai, India) yielding specific activity of spectrin_N_ 1.96×10^6^ cpm/µg and of spectrin_VL_ 1.8×10^6^ cpm/µg respectively. Fixed concentrations of ^125^I-spectrin_VL/N_ were incubated separately with Sepharose/agarose bound lectins (25 µl bead volume) of different sugar-linkage specificity. Con A (specific for α-Man and α-Glc), RCA (specific for β-D-Gal (GalNAc, β-Gal)), HPA (specific for α-or β-D-GalNAc), UEA (specific for α-L-Fuc) were used to demonstrate the presence of *N*-glycosylation. Similarly Jacalin (specific for β1,3GalNAc) and DBA (specific for α-GalNAc) were used to illustrate the presence of *O*-glycosylation. The lectins WGA (specific for GlcNAc and Neu5Ac), SNA, MAA, Achatinin-H (specific for 9-*O*-acetyl sialoglycosyl residue) were used to show the presence of sialic acids. Unbound radioactivity was removed using TBS-bovine serum albumin (1 mg/ml) and bound ^125^I-spectrin_VL/N_ was measured by Gamma-counter (Electronic Corporation, India) [47].

#### Identification of sialoglycopeptides from spectrin_VL_ containing α2,6 and α2,3 linked sialic acids

Purified spectrin_VL_ (150 µg) was run in 5% SDS-PAGE and α-spectrin & β-spectrin bands were digested partially by trypsin (200 ng) using in-gel trypsin digestion kit (Pierce, Rochford, USA) following the manufacturer's protocol. Digested and extracted tryptic fragments were dried under speed-vac and redissolved in 0.1% TFA (100 µl). An aliquot of the redissolved tryptic fragments were seperated on 7.5%–15% gradient SDS-gel. Remaining portion was neutralized by Tris-HCl (pH 8.0) and an aliquot was incubated separately with SNA-agarose (10 µl) and MAA-agarose (10 µl) for overnight at 4°C under mild shaking. The mixture was centrifuged at 5000 rpm for 10 min, supernatant collected as unbound fraction. Pellet was suspended in cold phosphate buffered saline (0.02 M, pH 7.0) and centrifuged at 5000 rpm for 10 min. Washed pellet was boiled with SDS-PAGE sample buffer, centrifuged at 5000 rpm for 5 min and the supernatant was ran in 7.5%–15% gradient gel.

#### TLC

Glycosidically bound sialic acids were released from purified spectrin_VL_ (60 µg) by hydrolysis with propionic acid (4 M) for 4 h at 80°C. Liberated SA was subsequently passed onto Dowex 50WX8 (100–200 mesh) cation and Dowex 2×8 (200–400 mesh) anion exchange columns. Free sialic acids eluted from these columns were separated on TLC plates (Merck KGaA, Germany) in 1-propanol/H_2_O (7∶3 v/v) and developed by spraying with orcinol/HCl/FeCl_3_ with heating at 180°C for 20 min [8], [26]. Commercially available sialic acids (Neu5Ac, Sigma) along with those released from BSM served as standards. In parallel, sialic acids released from spectrin_N_ were similarly processed.

#### Fluorimetric HPLC

An aliquot of liberated sialic acids was derivatized with 1,2-diamino-4,5-methylenedioxybenzene (DMB) and DMB-SA was separated on an RP-18 column (LichroCART 125-4 HPLC-cartridge, 5 µm; Merck, Germany) and detected at excitation and emission wavelengths of 373 nm and 448 nm respectively as described elsewhere [8], . In parallel, sialic acids purified from BSM were run for comparison.

#### MALDI-TOF MS

Each fraction corresponding to different forms of sialic acids was collected from fluorimetric HPLC and subsequently analyzed by MALDI-TOF-MS (Applied Biosystem, USA) using DHBA as matrix as described previously [8], [26], [48]. Positive ion mode was used for analysis. The acquired spectra were accumulations of 1000 laser shots.

### Molecular modelling of spectrin

#### Prediction of glycosylation sites

The protein sequences of human erythrocytic α- & β-spectrin were collected from NCBI database (gi: 119573202 and gi: 67782321 respectively). Prediction of *N*-glycosylation sites were performed with NetNGlyc 1.0 server (http://www.cbs.dtu.dk/services/NetNGlyc/). Predictions of *O*-glycosylation sites were done with NetOGlyc 3.1 server (http://www.cbs.dtu.dk/services/NetOGlyc/) [49]. Probable glycosylation sites above a threshold value of 0.35 were selected for solvent accessibility calculation.

#### 3-D structural modelling of spectrin modules

3D structure of the modules containing the potential glycosylation sites were modelled using Swiss Model software [50]. The quality of the models was validated using Structural Analysis and Verification Server (http://nihserver.mbi.ucla.edu/SAVES/).

#### Solvent accessibility of probable glycosylation sites

Solvent accessibility surface area of all amino acids residues of the models were calculated using ACCESS software [51]. The probable *N*- and *O*- linked glycosylation sites were identified by their percentage of surface exposure. Sites falling within the identified segments of α- and β-spectrin sequences by MASCOT program with significantly high intensity were eliminated from the list of probable sites as their non-glycosylated status was confirmed.

#### Attachment of carbohydrates to probable glycosylation sites

The structures of the modules attached to the carbohydrate at assigned Asn and Thr of the identified *N*- & *O*- glycosylation sites were optimized using molecular modelling software suite InsightII (2005) of Accelrys (San Diego, CA) by repeated energy minimization and molecular dynamics simulations with DISCOVER module. Energy minimization was performed alternatively with steepest descent and conjugate gradient methods (200 steps each using cff91 force field). Molecular dynamics simulation run was done with 10,000 steps of 1 fs after 1000 steps of equilibration with a conformation sampling of one in 100 steps at 300 K. At the end of the molecular dynamics simulation, the lowest potential energy conformation was picked using ANALYSIS module of Insight II for further energy minimization. The molecular dynamics simulation followed by energy minimization was performed on the glycosylation site residues attached with the sugar moiety while keeping the rest of the protein molecule fixed by applying positional constraints. This process was continued until satisfactory conformational parameters were achieved [26].

### Physicochemical studies

#### CD spectra of spectrin

Far-UV CD spectra (between 190 nm and 250 nm) measurement of equal amount (0.05 µg/µl) of spectrin_VL_ and spectrin_N_ were performed at 25°C on a JASCO J-715 spectropolarimeter using a quartz cuvette of path length 1 mm under continuous flush of nitrogen gas. The spectra shown are the average of ten data collected in continuous scan mode. The individual secondary structural contents of α-helix, β-sheet, and random coil were analyzed from the far-UV CD spectra using the K2D2 software [52].

#### Preparation of spectrin-depleted inside-out vesicles (IOVs) and binding with ^125^I-spectrin_VL/N_

Ghosts from RBC_N_ were incubated for 30 min at 37°C in 30 vol of EDTA (0.25 mM), PMSF (25 µg/ml), pH 8.0, centrifuged at 50,000 g for 25 min and the inside-out vesicles (IOVs) were suspended in the buffer containing sodium phosphate (10 mM), KCl (130 mM), NaCl (20 mM), EDTA (1 mM), NaN_3_ (0.5 mM), DTT (1 mM), pH 7.5 (Buffer A). Spectrin-depleted-IOV_N_ was stored at a concentration of 1 mg/ml for overnight at 4°C [53].

To demonstrate the binding of spectrin_N_ with spectrin-depleted-IOV_N_, ^125^I-spectrin_N_ (0–10 µg/ml) were incubated for 90 min at 0°C in a buffer A (100 µl) containing 20 µg/ml spectrin-depleted-IOV_N_ protein [53] and centrifuged at 50,000 g for 25 min at 4°C. Membrane-bound ^125^I-spectrin was washed with Buffer A and the radioactivity was counted by a Gamma-counter (Electronic Corporation, India). Nonspecific binding at each ^125^I-spectrin_N_ concentration was determined by the use of heat-denatured (70°C, 15 min) spectrin, and this value (10–28% of total counts) was routinely subtracted [53].

#### Estimation of sialic acid (SA) in serum

Estimation of total SA in serum was carried out colorimetrically by the thiobarbituric acid method after hydrolysis with 0.1 N sulfuric acid at 80°C for 1 hr [54]. The absolute value of sialic acid in serum was obtained from standard curve of authentic Neu5Ac.

Results are expressed as means ± S.D for individual sets of data. Each experiment was performed at least 3 times.

## Supporting Information

Figure S1 **Sequence of α-spectrin and β-spectrin with the identified and annotated fragments in red and the sequenced fragments underlined.**
(TIF)
