# Supplementary figures and images for: Glycosylation of Erythrocyte Spectrin and Its Modification in Visceral Leishmaniasis
Source: PLoS One. 2011 Dec 2;6(12):e28169. doi: 10.1371/journal.pone.0028169 (PMC3229537; doi:10.1371/journal.pone.0028169)

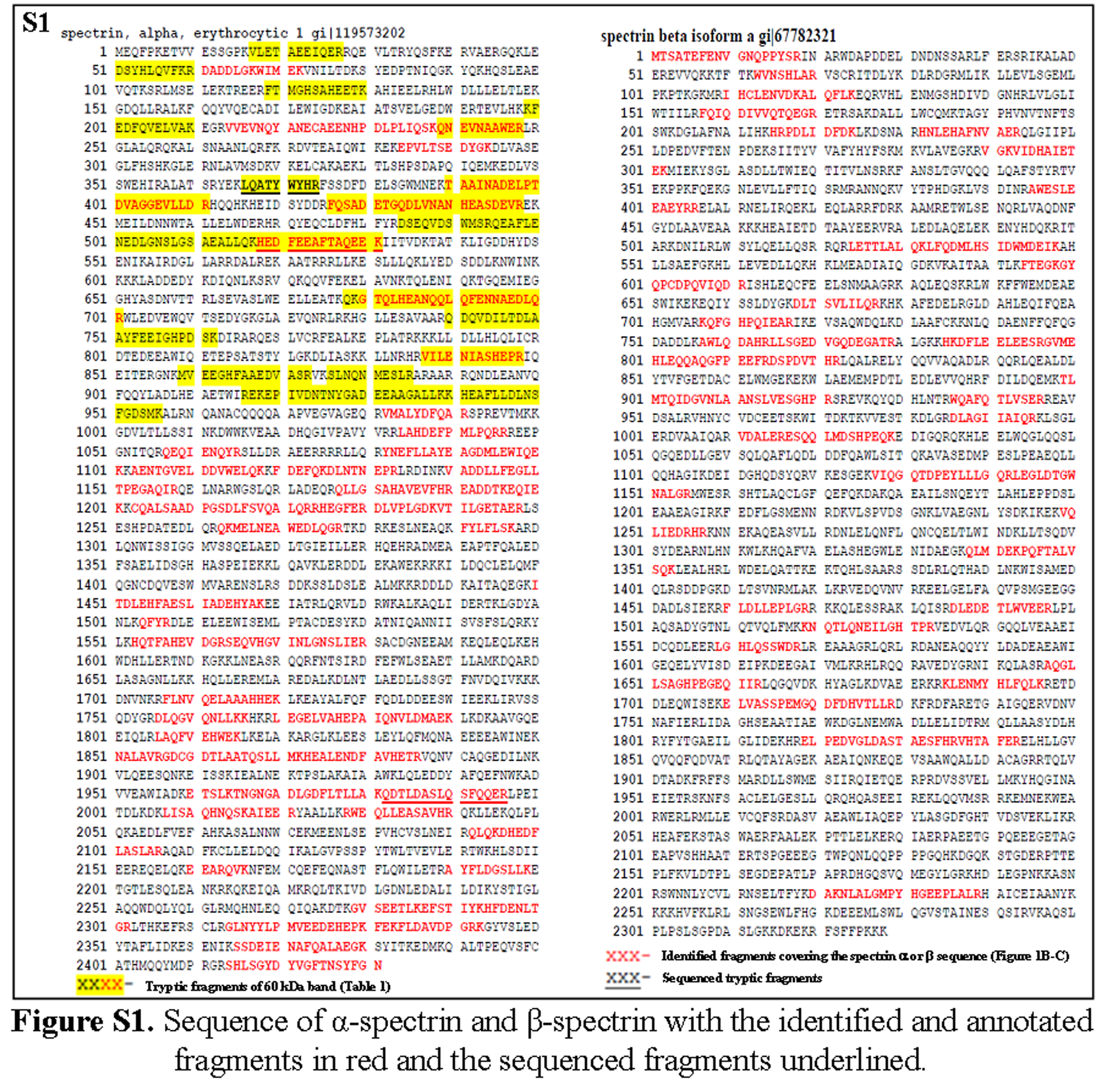

Supplement: Figure S1 — Sequence of α-spectrin and β-spectrin with the identified and annotated fragments in red and the sequenced fragments underlined. (TIF) [file pone.0028169.s001.tif]
